# Tregitope Peptides: The Active Pharmaceutical Ingredient of IVIG?

**DOI:** 10.1155/2013/493138

**Published:** 2013-12-25

**Authors:** Anne S. De Groot, Leslie Cousens, Federico Mingozzi, William Martin

**Affiliations:** ^1^Institute for Immunology and Informatics, University of Rhode Island, Providence, RI, USA; ^2^EpiVax, Inc., 146 Clifford Street, Providence, RI, USA; ^3^Genethon, Evry, France; ^4^University Pierre and Marie Curie, Paris, France

## Abstract

Five years ago, we reported the identification and characterization of several regulatory T-cell epitopes (now called Tregitopes) that were discovered in the heavy and light chains of IgG (De Groot et al. Blood, 2008). When added ex vivo to human PBMCs, these Tregitopes activated regulatory T cells (Tregs), increased expression of the transcription factor FoxP3, and induced IL-10 expression in CD4^+^ T cells. We have now shown that coadministration of the Tregitopes in vivo, in a number of different murine models of autoimmune disease, can suppress immune responses to antigen in an antigen-specific manner, and that this response is mediated by Tregs. In addition we have shown that, although these are generally promiscuous epitopes, the activity of individual Tregitope peptides is restricted by HLA. In this brief report, we provide an overview of the effects of Tregitopes in vivo, discuss potential applications, and suggest that Tregitopes may represent one of the “active pharmaceutical ingredients” of IVIg. Tregitope applications may include any of the autoimmune diseases that are currently treated almost exclusively with intravenous immunoglobulin G (IVIG), such as Chronic Inflammatory Demyelinating Polyneuropathy (CIDP) and Multifocal Motor Neuropathy (MMN), as well as gene therapy and allergy where Tregitopes may provide a means of inducing antigen-specific tolerance.

## 1. Introduction

In recent work [1], we identified an important trigger for the expansion and activation of regulatory T cells (Tregs), which are T cell epitopes contained in the framework sequences of immunoglobulin G (IgG). Further studies suggested that these peptides were natural T regulatory cell epitopes (Tregitopes) that may explain, at least in part, the tolerance-inducing effects of polyclonal immunoglobulin when delivered as a therapy (intravenous immunoglobulin or IVIG). The defining characteristics of Tregitopes were that they (i) stimulated CD4^+^, CD25^hi^, and FoxP3^+^ T cells; (ii) suppressed effector T-cell responses to other antigens in suppressor assays; and (iii) were associated with T cell IL-10 production in vivo and in vitro [1]. Subsequently, Tregitope peptides have been shown to replicate the effects of IVIG in mouse models of Multiple Sclerosis (EAE), allergy, and asthma, confirming our primary observations [2–4]. Consistent with their intrinsic immunosuppressive property, Tregitope peptides administered in complete or incomplete Freund's Adjuvant (CFA or IFA) suppress immune responses to coadministered antigens, but are not immunogenic per se [5].

While coadministration of Tregitope peptides with target antigen(s) effectively suppresses antigen-specific immune responses [6], Tregitope peptides are also particularly active in animal models on their own, if they are given during the acute phase of inflammation. For example, upon onset of diabetes Tregitope peptides formulated in IFA and delivered as a single dose to NOD mice (intraperitoneally) effectively suppressed diabetes in 58 percent of the mice for 25 weeks [6]. In a rigorous and independent NOD study conducted by the National Institute of Diabetes and Digestive and Kidney Diseases (NIDDK), Tregitope peptides were the only novel therapy of six tested that exhibited “notable trends;” three diabetic mice remitted entirely, all of which were in the Tregitope peptide treatment groups. None of the other therapies tested (such as DT22669 (DiaKine), Aralast NP (Baxter Healthcare), ISO-092 (Feinstein Institute for Medical Research), Celastrol (Pi and Pi Technology), and PGC-GLP-1 (PharmaINCorporation)) resulted in the prolonged remission seen with Tregitope peptides in this aggressive model of diabetes [7]. In addition, Tregitope peptides delivered in adeno-associated virus (AAV) ten days prior to TNBS (2,4,6 trinitrobenzenesulfonic acid-induced colitis) treatment were sufficient to suppress inflammatory bowel disease (and induce immigration of Tregs to the intestine) in this model of autoimmune disease [8].

## 2. Tregitopes: What Are They?

### 2.1. Natural and Induced Regulatory T Cells and Tolerance

It has become increasingly clear that CD4^+^CD25^+^FoxP3^+^ Tregs are an important component of immune regulation [9]. Autoreactive T cells with moderate T-cell receptor affinity may escape deletion in the thymus to circulate where they function as “natural” regulatory T cells (nTregs) [10]. Two distinct Treg subsets are described in the literature: natural nTregs specific for self-epitopes and generated by high-avidity selection in the thymus, and inducible iTregs that are derived from conventional (CD4^+^, FoxP3^−^) T cells following stimulation in the periphery [11, 12]. nTregs can induce the conversion of conventional T cells to iTregs via cytokine-dependent and -independent mechanisms, a process called infectious tolerance [13, 14].

It has been surmised that autologous proteins contain nTregitopes; however, few of these have been mapped. Immunoglobulin G has been known to exhibit tolerogenic properties for decades, and a number of previous publications have alluded to the potential presence of regulatory or “suppressor” epitopes in IgG constant domains, whether located to the constant (Fc) or binding region (Fab). For example, Baxevanis et al. described a tolerizing effect of Ig Fc that was localized to the CH2 region, consistent with the location of several Tregitopes [15]; a peptide isolated from the Fab region of an anti-idiotypic peptide (which overlaps one of the subsequently identified Tregitopes) suppressed Systemic Lupus Erythematosis in humans and in mice [16, 17]; a peptide derived from the highly conserved J region framework was shown by Warnke et al. to induce Tregs to expand and suppress immune responses in suppressor assays [18].

We perform immunogenicity studies for a range of clients in the preclinical phase of monoclonal antibody development [19, 20]. Using immunoinformatics tools (EpiMatrix [21]), now comprised in the ISPRI toolkit [20], we scanned the constant domains of human IgG and found that these and other regions of immunoglobulin G contained putative epitopes that were predicted to bind to more than one HLA and that were highly conserved in existing databases of IgG sequences. We hypothesized that they would serve to induce Tregs rather than T effector T cells, and that this might explain why some monoclonal antibodies that contain “foreign” (not seen in thymic development) sequences might not generate immune responses. Whether these Tregitope peptides induce nTregs or iTregs (peripheral, inducible Tregs), *or both*, remains to be determined. Nonetheless, we note that monoclonal antibodies that contain the full complement of Tregitopes are less likely to trigger immune responses despite hypervariable sequences that are comprised of de novo sequences and thus might appear “foreign” [22].

### 2.2. Tregitopes Are Found in IgG and in Fab as well as in Fc

As shown in Table 1, Tregitopes are promiscuous epitopes (as predicted by EpiMatrix) and are located in the Fc and the Fab regions of IgG. In contrast, Tregitopes are not found in other antibody isotypes (IgE, IgA, and IgM). It is interesting to note that IgG but not IgM was found to induce tolerance in seminal studies of tolerance to haptens, performed by Golan and Borel [23]. Tolerance induction to IgG Fc-conjugated antigens has been extensively described by Scott et al. [24–26], among others [27], who very clearly demonstrated that HLA class II was involved [28] and that the effect did not require Fc receptor binding [29], which supports our hypothesis that tolerance is induced by the presence of HLA class II-restricted Tregitopes. As described above, Tregitopes are also found in IgG Fab, which can explain why Fab is as effective as IgG in inducing Tregs [30]. The Tregitope hypothesis is distinct from the work of Anthony et al. [31], who suggest IVIG-induced tolerance is mediated through sialylated Fc that initiate an anti-inflammatory signaling cascade through the lectin receptor SIGN-R1 or DC-SIGN. Tregitopes may, however, explain the importance of binding to DC-SIGN, since that surface molecule traffics bound antigens directly to the class II processing and presentation pathway in dendritic cells.

### 2.3. Are Tregitopes the “Active Pharmaceutical Ingredient (API)” of IVIG? 

**Figure 1:**
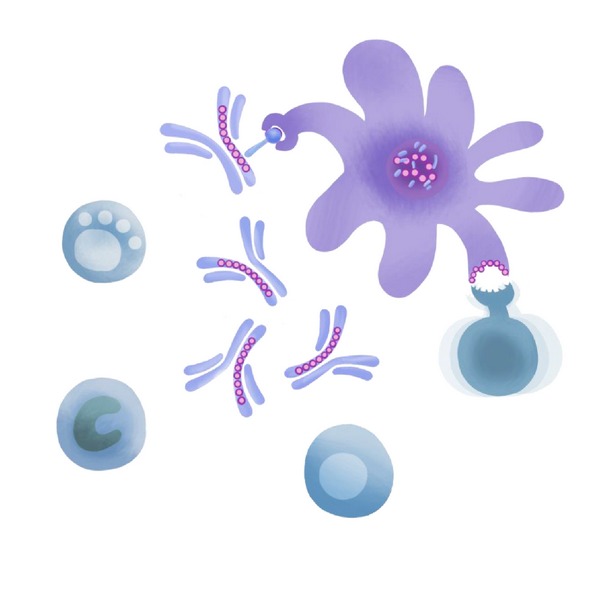
Potential IgG (and Tregitope) Mechanisms of Action. From left to right, IVIG has been demonstrated to affect the cells of the innate and adaptive immune system including NK cells, macrophages, B cells, T cells, dendritic cells and other antigen presenting cells.

The effects of IVIG have been attributed to a wide array of mechanisms (Figure 1). Additional mechanisms include the formation of immune complexes [32]; interaction of sialylated Fc with a novel macrophage receptor DC-SIGN [33]; blockade of Fc receptors leading to clearance of anti-self antibodies [34]; immunomodulation via anti-idiotypic interactions [35]; inhibition of complement-mediated tissue damage [36]; direct modulation of cytokine expression by leukocytes and endothelial cells; inhibition of superantigen-mediated T-cell activation [31, 37, 38]; and induction of nTregs [11]. IVIG has recently been shown to be associated with modulation of the regulatory T-cell axis, reduction of IL-17 [39], and enhancement of the suppressive function of Tregs [40]. In more recent studies by Massoud et al., the induction of Tregs by IVIG was shown to be dependent on IgG binding to a surface receptor called DCIR, followed by internalization and processing [41]. Many of these observed effects are consistent with the proposed Tregitope mechanism of action, as Tregitopes are peptides that would result from IgG internalization and processing, and have been associated with expansion of existing Tregs and induction of iTregs. Internalization of IgG (from IVIG), presentation of Tregitopes in the context of MHC class II, and expansion of Tregitope-specific Tregs would be consistent with recent observations that administration of IVIG induces expansion of Tregs and IL-10 secretion in vivo, in animals and in humans [9, 11, 42, 43].

## 3. Overview of Recent Tregitope Studies

### 3.1. Proposed Mechanism of Action

Since our original description of Tregitopes in 2008, we have significantly advanced our understanding of the Tregitope mechanism of action, leading to our working hypotheses: (1) Tregitope effects are contingent upon major histocompatibility complex (MHC) class II-mediated presentation to a T cell by an antigen-presenting cell (APC), (2) Tregs recognize Tregitopes presented in the context of MHC II and are activated, (3) these activated Tregs produce IL-10 and interact with the APC to reinforce the development of a tolerogenic phenotype, and (4) these tolerogenic APCs and/or Tregs act on adjacent antigen-specific effector T cells to suppress their effector responses and induce antigen-specific Tregs. Published studies on Tregitopes by our own group and our collaborators corroborate the proposed mechanism, including in vivo studies, a few of which are summarized in Section 4.

### 3.2. Comparison of Tregitopes to IVIG in EAE

In separate in vivo and in vitro studies reviewed previously [4], we collaborated with the laboratory of Khoury and Elyaman to compare Tregitope peptide treatment to IVIG in the EAE model of Multiple Sclerosis.The in vivo study evaluates the capacity of IgG-derived Tregitope peptides to generate antigen-specific adaptive tolerance induction to MOG35-55 epitopes. In that study, mice were presensitized by MOG immunization. EAE disease was established and treated with IVIG or human IgG Tregitope peptides 167 and 289 in saline [4]. A tolerogenic effect of Tregitope peptides on immune responses to the MOG35-55 epitopes was observed in vitro and in vivo. These results are consistent with previous reports by Legge et al. [44] who showed induction of Tregs by IgG fusion proteins with multiple sclerosis antigens.

### 3.3. Tregitope Peptides Suppress CD4^+^ T-Cell Responses and Are Not Immunogenic per se

In a recent paper, we demonstrated that Tregitope peptides are not immunogenic in vivo even when emulsified with potent adjuvants, such as IFA or CFA [5]. Moreover, in vivo administration of Tregitope peptides with IFA or CFA does not induce Th1 or Th2 cytokine expression under restimulation conditions in vitro. We investigated tolerance induction by codelivering Tregitope peptides with OVA using B cells. When B cells were pulsed with OVA and Tregitope peptides and transferred into naïve mice, we found that cellular and humoral immune responses to the OVA were suppressed as a result of their ability to induce Tregs and the absence of immunogenicity in the context of strong adjuvants [5].

### 3.4. Tregitope-Induced Tregs Also Modulate CD8^+^ T-Cell Responses

Immune responses directed against viral capsid proteins constitute a main safety concern in the use of AAV as a gene transfer vector in humans. Using Tregitope peptides, we showed that it is possible to modulate CD8^+^ T-cell responses to several viral antigens in vitro. Incubation of peripheral blood mononuclear cells with Tregitope peptides and viral epitope peptides triggered proliferation of CD4^+^CD25^+^FoxP3^+^ T cells that suppressed killing of target cells loaded with MHC class I antigens in an antigen-specific fashion, through a mechanism that required cell-to-cell contact. Expression of a construct encoding for the AAV capsid structural protein fused to Tregitope peptides resulted in reduction of CD8^+^ T-cell reactivity against the AAV capsid following immunization with a capsid-expressing adenoviral vector. This was accompanied by increased frequency of CD4^+^CD25^+^FoxP3^+^ T cells in spleens and decreased inflammatory infiltrates in injected tissues. This study demonstrated the feasibility of modulating CD8^+^ T-cell reactivity to an antigen using Tregitopes.

## 4. Conclusion

### 4.1. Can Tregitope Peptides Replace IVIG?

IVIG is a pooled blood product from 10,000 or more donors. Many adverse effects (AEs) associated with IVIG administration are mild and transient, including headache, flushing, malaise, fever, chills, fatigue, nausea, vomiting, diarrhea, blood pressure changes, tachycardia, and anaphylactic reactions. Rare but more severe AEs include acute renal failure, thromboembolic events, skin-related effects including toxic epidermal necrolysis, and aseptic meningitis [45]. Many of these effects have been attributed to the targeting function of individual antibodies in the polyclonal immunoglobulin mixture. In general, however, IVIG is considered relatively safe. Thus, the main incentives to seek alternatives are either economic (to reduce the cost to patients and insurers) or to preserve IVIG supply for patients with primary immunodeficiencies who require the functional antibody component of IVIG to protect against infections. Tregitopes may, in part, explain the mechanism by which IVIG exerts its tolerogenic effect. Where those effects are Treg-mediated, Tregitopes might serve as an alternative IVIG for autoimmune conditions that may be safer (due to the absence of the functioning antibody component) and more effective.

If the induction of Tregs by IVIG can be attributed to Tregitopes, then a number of autoimmune diseases for which IVIG therapy is currently used, on-label and off, may be appropriate targets for immunomodulatory formulations that only contain Tregitope peptides. Two examples of disease in which IVIG is the predominant immunotherapy include Chronic Inflammatory Demyelinating Polyneuropathy (CIDP) and Multifocal Motor Neuropathy (MMN). Introduction of Tregitope peptides as alternatives to IVIG would also have a dramatic impact on the demand for IVIG for immune modulation therapy [11, 43, 45]. IVIG also acts rapidly and effectively in Immune Thrombocytopenic Purpura (ITP), Kawasaki Syndrome (KS), polymyositis, dermatomyositis, neurological syndromes such as Guillain-Barré and CIDP, cases of severe steroid-dependent asthma, and many others [46, 47]. Tregitope-peptide therapy may be an attractive alternative to the systemic immune suppression treatments that are sometimes used for these conditions.

### 4.2. Additional Tregitope Applications

**Figure 2:**
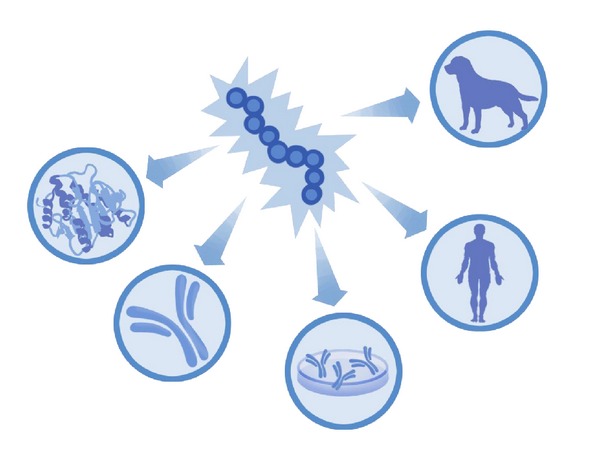
The broader relevance of Tregitopes is shown from left to right (counter clockwise). Tregitopes may be incorporated into protein drugs and monoclonal antibodies, to suppress antitherapeutic protein immune responses. Removing Tregitopes from autologous antigens would improve the ability of protein engineers to develop effective antibodies for target (autologous) antigens, and engineering Tregitope-depleted antibodies may improve the delivery of vaccine antigens. As described in this paper, Tregitopes may represent a new therapeutic option for autoimmune disease. In addition, Tregitopes could be used to suppress autoimmune diseases in companion animals.

Replacement of a pooled-donor, blood-derived product for which the mechanism of action is not certain with a therapeutic product for which the Active Pharmaceutical Ingredient (API) is precisely defined would represent a major step forward for the field of autoimmune disease therapy. In addition, since Tregitopes appear to be able to induce antigen-specific tolerance (induced Tregs that are specific to coadministered proteins), the possibility of tailoring the Tregitope therapy to specific autoimmune diseases would represent an additional advantage for clinical applications (Figure 2). One could imagine allergen-specific treatments using combinations of Tregitope peptides and allergen proteins or peptides, and treatment for autoimmune diseases such as diabetes, which would rely on coadministration of Tregitope peptides and diabetes antigens. The idea of specifically generating MOG-reactive Tregs is also particularly attractive for the therapy of Multiple Sclerosis where, based on our data in the EAE mouse model, we believe that Tregitope peptides induce antigen-specific adaptive tolerance. Tregitopes may also have even broader applications in protein therapeutics, animal health, and blood factor or enzyme replacement therapy.

Treatment of many autoimmune diseases relies on immunosuppressive therapy rather than on treatments directed toward restoring a balance between effector and regulatory immune responses. Given that Tregitope peptides appear to induce adaptive tolerance in a mouse model, the next consideration is to evaluate the optimal formulation and determine the best time course of the tolerance induction, in addition to measuring the duration of response, the dose required, the safety and toxicity of the treatment (vis-à-vis other immune responses), and optimal formulation/route of administration. Our preliminary studies suggest that adaptive tolerance induction may be within reach, raising hopes that we are on the right path for the development of an effective immunotherapy-based approach to autoimmune disease.

## Figures and Tables

**Table 1:** Partial list of previously identified human IgG tregitopes.

	HLA		Location	In vitro validation		In vivo validation	
	Promiscuous	EMX score*	IgG	Murine	Human	Mouse	Human
Tregitope 167	Y	30.05	Fc	Y	Y	Y	In Fc
Tregitope 289	Y	22.57	Fc	Y	Y	Y	In Fc
Tregitope 084	N	14.07	Fab	(N/A)	Y	—	∗∗
Tregitope 009	N	14.09	Fab	(N/A)	Y	—	∗∗
Tregitope 029B	N	16.38	Fab	(N/A)	Y	Y	Y
Tregitope 134	N	2.70	Fab	(N/A)	Y	Y	—

*EMX Score: EpiMatrix-predicted MHC binding promiscuity; correlates with T-cell response.

N/A: no murine homolog.

**Other data, not able to disclose.

In Fc: fc Fusion proteins have both Tregitope 167 and 289 present. See D. W. Scott publications.

## Glossary

Tregs: Regulatory T cells
Tregitopes: Treg epitopes (first described for IgG in 2008)
nTregs: Natural T regulatory cells
iTregs: Inducible T regulatory cells.
